# [Retracted] EGFR expression is associated with cytoplasmic staining of CXCR4 and predicts poor prognosis in triple-negative breast carcinomas

**DOI:** 10.3892/ol.2026.15503

**Published:** 2026-03-02

**Authors:** Rong-Hui Li, Wen-He Huang, Jun-Dong Wu, Cai-Wen Du, Guo-Jun Zhang

Oncol Lett 13: 695–703, 2017; DOI: 10.3892/ol.2016.5489

Following the publication of this article, an interested reader drew to the authors' attention that certain of the mouse images shown in Fig. 3A on p. 700 appeared to be strikingly similar, such that a pair of the model mice may have been shown to represent different experimental conditions. In addition, it subsequently came to light that a pair of tumor images featured for the shCXCR4-2# and sh-1#-2# experiments were remarkably similar concerning certain of their features, even though the image had apparently been truncated as far as its representation in the latter experiment was concerned.

Regrettably, the authors were not able to identify the original images given the time that has elapsed since the publication of this article and changes in the personnel in the laboratory. Although the possibility of issuing a Corrigendum was considered, the Editor of *Oncology Letters* has decided that this paper should be retracted from the Journal on account of a lack of confidence in the presentation of the data in Fig. 3. The authors were asked for an explanation to account for these concerns, but the Editorial Office did not receive a satisfactory reply. The Editor apologizes to the readership for any inconvenience caused.

